# Rabbit Achilles tendon full transection model – wound healing, adhesion formation and biomechanics at 3, 6 and 12 weeks post-surgery

**DOI:** 10.1242/bio.020644

**Published:** 2016-09-15

**Authors:** Gabriella Meier Bürgisser, Maurizio Calcagni, Elias Bachmann, Gion Fessel, Jess G. Snedeker, Pietro Giovanoli, Johanna Buschmann

**Affiliations:** 1 Division of Plastic Surgery and Hand Surgery, University Hospital Zurich, Sternwartstrasse 14, Zurich 8091, Switzerland; 2 Uniklinik Balgrist, Department of Orthopedics, Forchstrasse 340, Zurich 8008, Switzerland; 3 Laboratory for Orthopaedic Biomechanics, Swiss Federal Institute of Technology in Zurich (ETHZ), Rämistrasse 101, Zurich CH-8092, Switzerland

**Keywords:** Achilles tendon, Adhesion, Load until failure, Ultimate stress, Elastic modulus

## Abstract

After tendon rupture repair, two main problems may occur: re-rupture and adhesion formation. Suitable non-murine animal models are needed to study the healing tendon in terms of biomechanical properties and extent of adhesion formation. In this study 24 New Zealand White rabbits received a full transection of the Achilles tendon 2 cm above the calcaneus, sutured with a 4-strand Becker suture. Post-surgical analysis was performed at 3, 6 and 12 weeks. In the 6-week group, animals received a cast either in a 180 deg stretched position during 6 weeks (adhesion provoking immobilization), or were re-casted with a 150 deg position after 3 weeks (adhesion inhibiting immobilization), while in the other groups (3 and 12 weeks) a 180 deg position cast was applied for 3 weeks. Adhesion extent was analyzed by histology and ultrasound. Histopathological scoring was performed according to a method by Stoll et al. (2011), and the main biomechanical properties were assessed. Histopathological scores increased as a function of time, but did not reach values of healthy tendons after 12 weeks (only around 15 out of 20 points). Adhesion provoking immobilization led to an adhesion extent of 82.7±9.7%, while adhesion inhibiting immobilization led to 31.9±9.8% after 6 weeks. Biomechanical properties increased over time, however, they did not reach full strength nor elastic modulus at 12 weeks post-operation. Furthermore, the rabbit Achilles tendon model can be modulated in terms of adhesion formation to the surrounding tissue. It clearly shows the different healing stages in terms of histopathology and offers a suitable model regarding biomechanics because it exhibits similar biomechanics as the human flexor tendons of the hand.

## Abstract (precis)

**Summary:** The rabbit Achilles tendon full transection model can be used to study adhesion extent in a controlled way. It also mimics the biomechanics of human hand flexor tendons.

## INTRODUCTION

Plastic surgeons as well as trauma surgeons dealing with lacerated tendons are confronted with two main problems: re-rupture and adhesion formation (Elliot and Giesen, 2013). The re-rupture is often caused by an insufficiently strong scar tissue lacking the aligned collagen organization seen in normal healthy tendons (Galatz et al., 2015). Adhesion between the healing tendon and the surrounding tissue in the early healing phase up to 6 weeks (Wu and Tang, 2013a,b) occurs in 7 to 15% of the cases leading to increased work disability (Tang, 2005).

Hence, in order to develop new suture techniques to enhance strength at the repair site (Buschmann et al., 2011; Haimovici et al., 2012) as well as biological (Crispim et al., 2015), cellular (Zhang et al., 2015), chemical (Zhao et al., 2013) or material-based (Sawadkar et al., 2014) strategies to overcome the formation of fibrous scar-like tissue and adhesion to the surrounding tissue, adequate non-murine animal models are needed to get insight into the impact of such approaches. At best, the same animal model can be used for both the examination of a new strategy's impact on the biomechanical strength as well as on the adhesion formation. A full transection tendon model with similar biomechanics as the human flexor tendons and a well-controlled adhesion induction would serve this need because full laceration injuries commonly are accompanied by scarry adhesions (Wu and Tang, 2013a,b).

Currently, small and large animals are being used addressing tendon healing. For example, the murine patellar tendon has been used to study spatiotemporal collagen gene expression, histology, and biomechanics following full-length injury (Dyment et al., 2012). Moreover, the rat supraspinatus model was used to assess the effects of gelatine hydrogel sheets having a growth factor incorporated, with histological and biomechanical readouts at different time points (Tokunaga et al., 2015). Compared to these rather small animals, larger animals like the rabbit do adequately represent not only histological changes during the healing process, but may also mimic the strength of human lacerated tendons, facilitating the translation from preclinical to clinical trials.

For example, the rabbit Achilles tendon (AT) has similar biomechanical properties as the human hand flexor tendon, exhibiting almost the same ultimate failure load (Goodman and Choueka, 2005). Although the AT is not an intrasynovial tendon like the flexor tendon, the peritenon that covers the AT is prone to develop adhesions to the surrounding tissue (Meier Buergisser and Buschmann, 2014; Meier Buergisser et al., 2014). Because of these two characteristics, the rabbit AT has been used in our group to study the effect of a synthetic polymer carrier device that was placed around the fully transected AT after conventional suture by evaluation of the cellular response towards the implant (Buschmann et al., 2013a,b). Furthermore, the rabbit AT is large enough to examine accurately morphological as well as cell-based tissue changes with non-invasive ultrasound (Buschmann et al., 2014), which might be limited in smaller animal models in terms of specimen size.

Hence, the rabbit Achilles full transection model is a good and suitable model to study tendon pathology. In the presented work, we used the rabbit AT model to measure baseline biomechanical properties, including load until failure, cross sectional area (CSA), stiffness, modulus of elasticity and failure stress as a function of time in order to provide a large animal model with similar biomechanical properties as the human hand flexor tendons. These properties were assessed 3, 6 and 12 weeks after full transection.

Furthermore, we developed two well-controlled adhesion-inducing casting regimens where adhesion formation is shown to be modulated by the ankle angle used during immobilization. The casting regimens were compared and adhesion extent evaluated by histology and dynamic ultrasound is presented for the 3 and 6 weeks post-surgery situations, where adhesion formation is usually highest over the entire healing time (Wu and Tang, 2013a,b). After 6 weeks, adhesions become generally more elastic and get easier to break (Wu and Tang, 2013a,b). Finally, also histopathological changes as a function of time were determined by scoring differently stained tissue sections and evaluation by a scoring system developed by Stoll and co-workers (2011).

## RESULTS

### Adhesion provoking and adhesion inhibiting immobilization

**Fig. 1.**
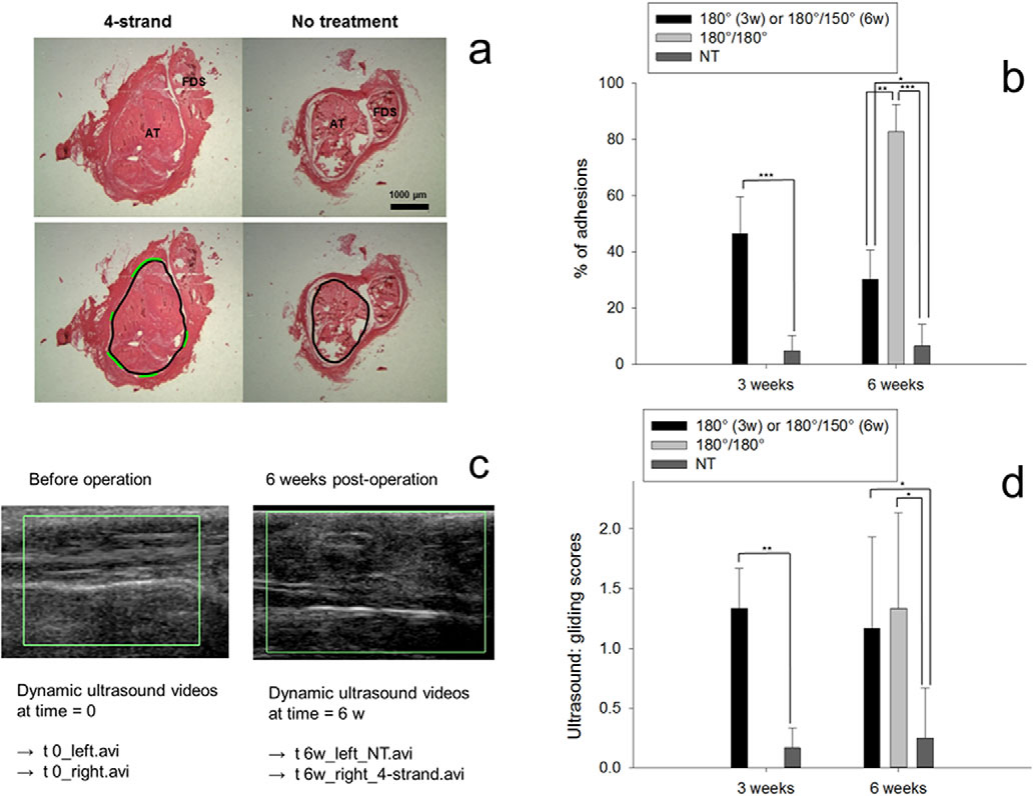
**Assessment of adhesion.** (A) Determination of adhesion extent based on Picrosirius Red stained section after a method by Tan et al. (2010) in five subsequent cross-sections, where the black line denotes the circumference of the Achilles tendon (AT) and the green line the zone of adhesion to the surrounding tissue. Flexor digitorum superficialis (FDS) is also shown. (B) Percentage of adhesions to the surrounding tissue based on histology, calculated by division of length (green line) by length (black line) in A. (C) Ultrasound references to Movies 2 and 4. (D) Gliding scores, with 0=good gliding (no adhesion), 1=middle gliding (some adhesion) and 2=no gliding (maximum adhesion) (see Movies 1–4). One-way ANOVA was conducted. Pairwise comparison probabilities (*P*) were calculated using the Fisher's PLSD *post hoc* test. **P <0.05. NT, no treatment (contralateral side); 180 deg/150 deg, immobilization protocol where the ankle angle of the cast was changed from 180 deg to 150 deg after 3 weeks; 180 deg/180 deg, immobilization protocol where the ankle angle of the cast was 180 deg during 6 weeks (cast renewed after 3 weeks). *n*=3 for 180 deg/180 deg; *n*=6 for 180 deg/150 deg; *n*=6 for NT group, at 3 and 6 weeks post-operation.

The adhesion extent was histologically assessed at 3 and 6 weeks post-surgery, where the legs of the rabbits had been immobilized with an angle of 180 deg in the 3-week group, while the 6-week group was divided into two subgroups subjected to different post-operative treatments (Fig. 1). The two different post-operative casting protocols had a significant impact on the adhesion extent. A change of the ankle angle from 180 deg to 150 deg after 3 weeks reduced the adhesion extent significantly from 82.7±9.7% to 31.9±9.8% (*P*<0.05, unpaired *t*-test). Compared to the situation at 3 weeks where adhesion had formed to an extent of 46.5±13%, the casting angle for the subsequent 3 weeks had a tremendous impact on the resulting adhesion. While adhesion was reduced when the angle was changed to 150 deg, it was significantly increased when the angle was kept constant although a new cast had been applied. Notably, also in the non-treated counter legs, adhesion formation was different for the 3-week and 6-week groups, though not significantly, with the not-treated (NT) specimen of the 6-week group showing a higher adhesion extent. Additionally, the gliding capacity was semi-quantitatively scored by dynamic ultrasound. The same trend as found for the adhesion extent was also determined for the gliding functionality.

### Biomechanics

**Fig. 2.**
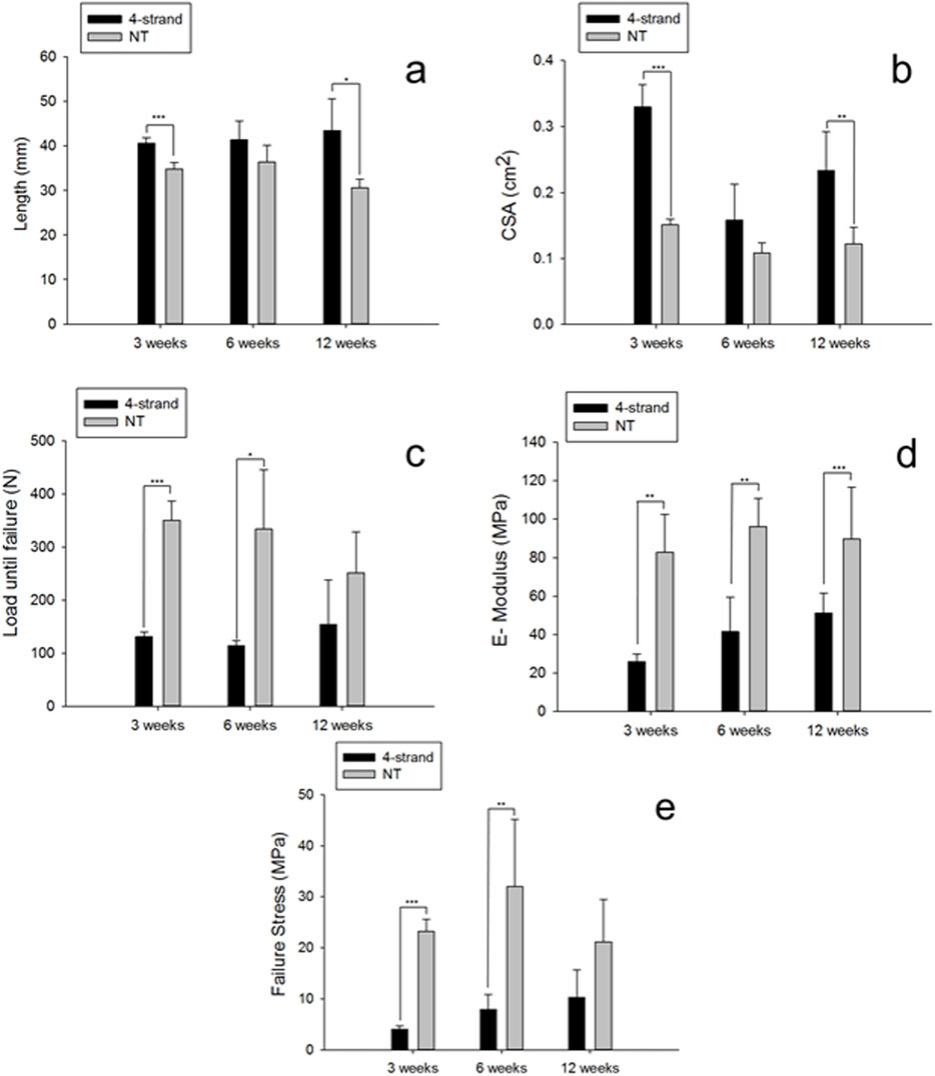
**Size and biomechanics.** Length (A), CSA (B), load until failure (C), E-Modulus (D) and failure stress (E) determined for the extracted Achilles tendons at 3, 6 and 12 weeks post-operation. For the 6-weeks group, the casting regime was 180 deg/150 deg (for further information, see Materials and Methods section). NT, no treatment (contralateral legs) except for time point 12 weeks where NT was based on rabbits not treated at all; 4-strand, 4-strand Becker suture. *P*<0.05; **P<0.01; ***P<0.001; unpaired *t*-test. *n*=3 for 4-strand group, *n*=6 for NT group at all time points.

Fig. 2 shows the biomechanical results at 3, 6 and 12 weeks post-surgery; for the 6-week group, the rabbits were casted with the 180 deg/150 deg regime (adhesion inhibiting model). The size was evaluated by length and cross-sectional area (CSA) of the specimen. In contrast to the non-treated Achilles tendons, the treated tendons were longer at all time points post-operatively (Fig. 2A). Moreover, they exhibited a larger CSA than the untreated tendons, which was clearly expressed at 3 weeks post-surgery; at this time point the tendons were swollen and had significantly higher CSAs than at 6 or at 12 weeks post-surgery (Fig. 2B).

In terms of strengths, the load until failure was lower for the operated tendons compared to the non-treated counter-legs at all time points; however, while the operated tendons had a significantly lower strength than the non-treated specimen at 3 and 6 weeks post-surgery, the difference was not significant anymore at 12 weeks (Fig. 2C). As for the elastic modulus, it was significantly reduced in case of operation; however, it significantly increased during the healing period from 3 to 12 weeks post-operation for operated legs (Fig. 2D). Finally, failure stresses of operated legs also increased with time, however not significantly, only as a tendency. At 3 and 6 weeks post-operation, treated legs exhibited a significantly lower failure stress compared to non-treated legs; at 12 weeks, no significant difference was found (Fig. 2E).

### Histopathological scoring

**Fig. 3.**
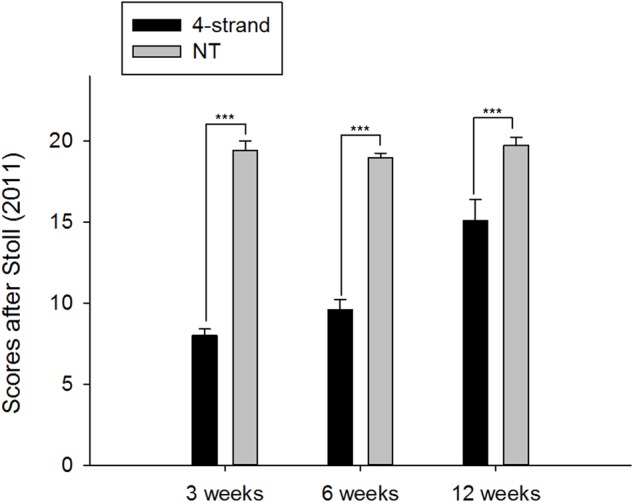
**Analysis of H&E- and AB-stained histological sections by the scoring system of** **Stoll et al. (2011****).** Sections were analyzed at 25× and 100× magnification. The system includes inspection of extracellular matrix organization of the whole repaired tendon (scores 0, 1 and 2), cellularity (scores 0, 1 and 2), cell alignment (scores 0, 1 and 2), cell nucleus morphology (scores 0, 1 and 2), cell distribution (scores 0 and 1), proteoglycan content (scores 0 and 1), organization of the tendon callus (scores 0, 1 and 2), configuration of callus (scores 0 and 1), integration of constructs to the normal tissue (scores 0, 1 and 2), vascularization (scores 0 and 1), metaplasia (scores 0, 1, 2 and 3) and features of inflammation (scores 0 and 1) (Table 1). All scores are summed up (maximum 20), with 0 scores standing for damaged tissue and 20 scores denoting healthy tissue. *P<0.05; **P<0.01; ***P<0.001; unpaired *t*-test. *n*=3 for 4-strand group, *n*=6 for NT group at all time points.

Histopathologically, a clear increase in scores after Stoll et al. (2011) was observed from time points 3 weeks to 6 weeks and to 12 weeks (Fig. 3). At all time points, operated legs showed a significantly lower score-sum when compared to their counter legs that were not treated.

**Table 1.**
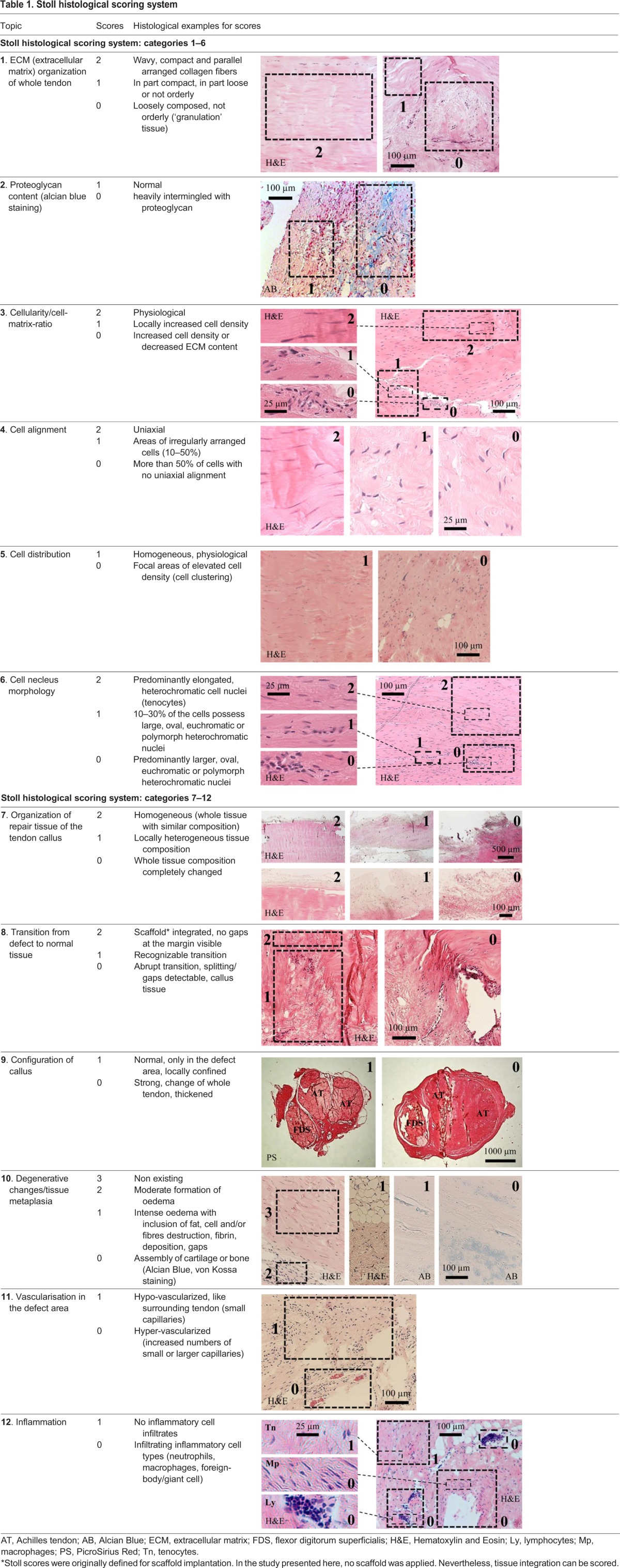
**Stoll histological scoring system**

## DISCUSSION

In contrast to other limbs of the musculoskeletal system the healing of lacerated tendons usually takes a long time because there is a lack of an inclusive vascular network, the tendon tissue is not densely populated by cells and the tenocytes are low in their metabolism, which together lead to a poor regenerative potential. Often, healing is accompanied by the development of a scar tissue and adhesions to the surrounding, restricting the gliding capacity and thus the range of motion. Furthermore, this scar-like fibrous tissue leads to poor mechanical properties, lower strength and lower elastic modulus, respectively. The two most common problems occurring after tendon injuries are re-ruptures and adhesion (Elliot and Giesen, 2013).

In order to improve tendon healing, i.e. to strengthen the tendon tissue at early time points as well as to reduce adhesion formation by supporting scar-less healing mechanisms (Galatz et al., 2015), proper animal models are needed. So far, when studying basic mechanisms of tendon healing as well as therapeutic agents that could be translated to clinical care, the rabbit model has been widely used (Thomopoulos et al., 2015). The rabbit Achilles tendon model has been reported to be beneficial when the effect of stem cell application was under view (Chong et al., 2007), when collagen implants were tested to replace large defects (Oryan et al., 2013), or when the influence of dehydration or irrigation was investigated in terms of adhesion formation (Saygi et al., 2012).

Also in our research group, the fully transected and sutured rabbit Achilles tendon model has been successfully used to study the cellular response towards a newly synthesized electrospun polymer tube that was placed around a conventionally sutured tendon to act as a physical anti-adhesion barrier (Buschmann et al., 2015). Furthermore, the rabbit Achilles full transection model can also be used in non-invasive longitudinal ultrasound studies, with Power Doppler assessments elucidating hematomas or increased vascularization at the repair site, and gray scale variation corresponding to histological changes on the collagen and cell level over time (Buschmann et al., 2014). In addition, although the Achilles tendon is not an intrasynovial tendon, adhesion formation to the surroundings (especially to the peritenon) can also be fairly studied in this model by histology (Tan et al., 2010), which has been successfully used to compare a conventionally 4-strand Becker sutured tendon with a physical barrier-bearing test group (Meier Buergisser et al., 2014). Hence, the full transection rabbit Achilles tendon model is a viable option to address both major problems, biomechanics similar to the human hand flexors and adhesion formation to the surrounding tissue.

In the work presented here, the histopathological status and the biomechanical properties at 3, 6 and 12 weeks post-operation are presented. Furthermore, two different post-operative casting regimens were compared in terms of adhesion formation to the surrounding tissue (an adhesion evoking and an adhesion reducing regimen) and the extent was assessed by dynamic ultrasound and histology at 3 and 6 weeks. Adhesion formation generally starts at 1.5 weeks, peaks at around 4 weeks and becomes more elastic and easier to break at approximately 7 weeks post-surgery (Wu and Tang, 2013a,b). Based on histology, a single recasting after 3 weeks changing the ankle angle from initial 180 deg to 150 deg significantly reduced the adhesion extent from 46±13% at 3 weeks to 30±10% at 6 weeks. In contrast, if the angle was kept constant at 180 deg, although the leg was freshly casted after 3 weeks, the adhesion significantly increased from 46±13% to 83±10% at 6 weeks. Continuing with these data, semi-quantitative scoring of functional gliding *in vivo* by dynamic ultrasound showed better gliding in the 180 deg/150 deg group compared to the 180 deg/180 deg group, although there was no significant difference. Such findings not only show the high importance of an accurate post-operative treatment, but they also offer options to study the effects of anti-adhesives in tendon healing (Meier Buergisser and Buschmann, 2014) under extreme conditions (referring to the adhesion evoking cast). Furthermore, these two different post-operative treatments can also be used to study scarry healing (adhesion evoking cast) in contrast to a more regenerative healing exhibiting less scar formation (adhesion reducing cast). Finally, the idea of recasting the Achilles tendon under different ankle angles could be extended, by further recasting steps after time periods shorter than three weeks and where the angles are set even smaller than 150 deg, thereby continuously loading and stretching the healing tendon in a controlled way and inhibiting adhesion formation due to positional changes within the cast.

When addressing the physical and biomechanical properties of the rabbit ATs, a significantly higher length for treated specimen compared to non-treated tendons was found for 3, 6 and 12 weeks post-surgery. The increased length of the healing tendons has an impact on the force transmission from the muscle to the calcaneus and on tendon functionality in general. As reported by Maquirriain, tendon lengthening is an important cause of morbidity and may produce permanent functional impairment (Maquirriain, 2011). Of course, longer tendons need a higher pre-tension to exhibit the same force as shorter tendons; moreover, anatomical changes also most often affect the muscle which gets shorter as the muscle needs a higher dorsal flexion in order to provide the necessary force for plantar flexion due to the longer tendon (Suydam et al., 2013). Tendon lengthening may be caused by gap formation, whereby the size of the gap is critical. When the gap was less than 3 mm in a canine flexor tendon model, the range of motion was not significantly reduced, however, when the gap was larger, the range of motion was seriously affected (Gelberman et al., 1999); therefore, not surprisingly we found that biomechanical properties of the significantly longer tendons also exhibited smaller failure stress values and smaller elastic moduli compared to untreated healthy tendons. Nevertheless, regarding changes over time, both parameters significantly increased from 3 to 12 weeks. As for the comparison of treated versus untreated tendons, there were significant differences in failure load and failure stress at 3 and 6 weeks, however, at 12 weeks (which was chosen as the longest time frame here), there was no statistically significant difference suggesting that reconstitution of two important biomechanical properties needed to prevent a re-rupture had been successful. Hence, this data set can be used as a time-dependent baseline set of values, especially when treatment protocols are evaluated that aim to improve strength of reconstructed tendons.

An interesting finding was the fact that the load until failure was slightly higher at 3 than at 6 weeks. This corresponds to other findings (Joshi et al., 2009) where this effect was explained on the basis of cellularity and vascularity. While cell density peaks at around 4 weeks post-operation in the healing tendon tissue, vascularity is highest at around 6 weeks, the latter being responsible for lower failure loads at 6 weeks compared to 3 weeks.

Reduced biomechanical properties may, however, not primarily be attributed to an increased length of the specimen. The tissue composition is also a key factor leading to impaired biomechanics, found here at 3 and 6 weeks during the healing process. When the histological sections were compared and scored after Stoll et al. (2011), there was a gradual and significant increase from 3 to 12 weeks post-operation, however, even at the latest time point there was no full recovery of the tissue, never reaching the full set of characteristics (although failure load and failure stress had recovered by then). Five features in particular did not reach the healthy status fully: characteristics concerning the cells such as the nucleus morphology, the tenocyte distribution, the cell alignment and the cell-to-matrix ratio as well as extracellular matrix (ECM) organization did often only reached 1 point from a range of 0–2 points, thereby lowering the sum of scores to around 15 points (from a maximum of 20) even 12 weeks post-operation. These characteristics concerning mostly cellular morphology, cell density and ECM would probably be fully reconstituted to a native level only after 12 weeks; the final modeling of the tendon tissue including collagen fiber alignment and decrease of cell density may take up to one year (Sharma and Maffulli, 2006).

Although the rabbit full transection Achilles tendon model acts as a suitable model (i) to mimic the biomechanics of ruptured human flexor tendons in the hand and (ii) to study adhesions because they are tunable by a controlled recasting regimen, there are also some potential complications that may arise. Anesthesia is delicate because rabbits may suddenly stop breathing, and isoflurane percentage has to be tuned accordingly. Moreover, the breathing should be controlled continuously in order to immediately be able to give support by hand. Another critical point is the cast: it should not be too tight because blood circulation may be hampered, especially at the margin to the toes (the cast should allow the toes to be moved a bit); however, it should not be too loose, as this may end up in gap formation at the repair site. Therefore only trained persons should make the cast.

## CONCLUSION

In sum, we here describe a full transection rabbit Achilles tendon model, serving as a suitable model for tendon healing. The model appropriately offers biomechanical readouts at any time point post-surgery similar to the human flexor tendons and simultaneously the option to study the adhesion extent: the two most important assessments for tendon rupture repair. Histological sections allow studies on inflammatory reactions towards implant materials and chemical cues to improve tendon healing, as well as towards cellular therapy. In addition, two different casting protocols were presented and their well-controlled impact on the adhesion formation was discussed. It was shown that by changing the ankle angle when recasting the tendon, the adhesion extent could be varied by 50%. This might be of high value when the adhesion extent should be modulated to a certain planned extent in order to study anti-adhesive agents and their effects under certain conditions.

## MATERIALS AND METHODS

### Animals

For this *in vivo* study, 24 female New Zealand White rabbits aged 12 to 16 weeks were used (Charles River, Research Models and Services, Germany). They were specific pathogen free (SPF). All animals were housed in pairs in two interconnected cages, each of them with a bottom area of 70 cm×70 cm and a height of 62 cm (Indulab, Switzerland). The animals were maintained under controlled conditions: temperature 22±1°C, 45% relative humidity, 15 air changes per hour and a light/dark rhythm of 12 h. The rabbits had free access to water (automatic water supply), autoclaved hay and straw *ad libitum* and to standard pellet diet (Kliba Nafag, Nr. 3410, Provimi Kliba AG, Switzerland). Ethical approval for the experiments was obtained from the veterinary office of Zurich, Switzerland (reference numbers 92/2009 and 193/2012). Prior to surgery, all animals were acclimatized to their environment for 2 weeks.

### Achilles tendon repair

**Fig. 4.**
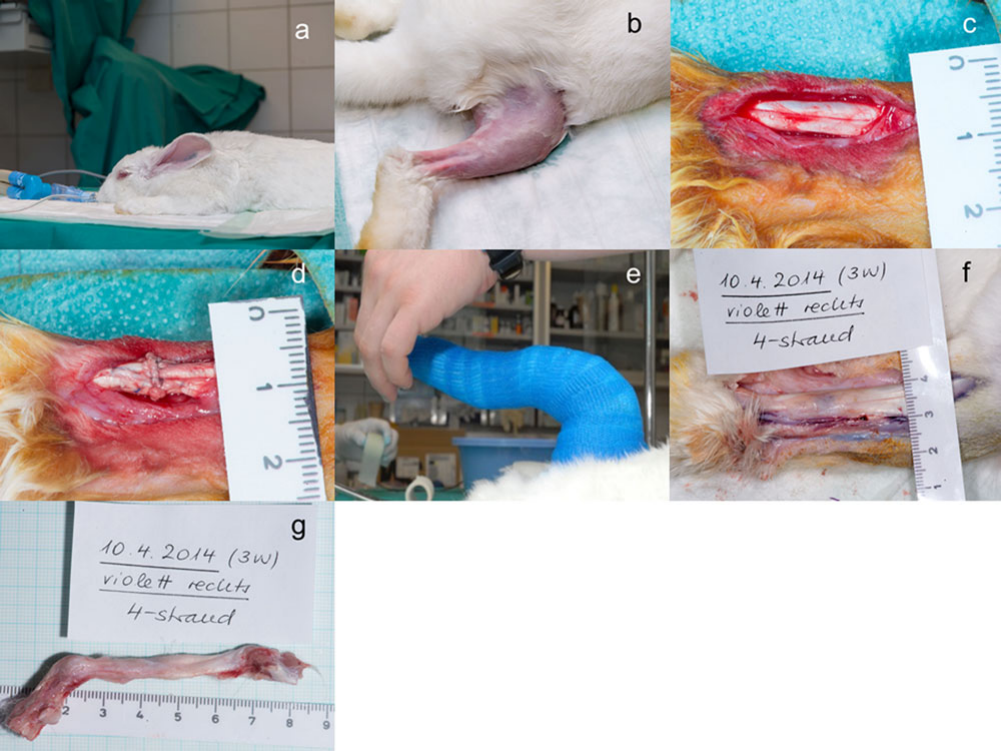
**Experimental procedure for rabbit Achilles tendon (AT) repair.** (A) Rabbit receiving isoflurane anesthesia. (B) Shaved rabbit hind leg ready to be operated. (C) Rabbit AT exposure (just before transection). (D) Rabbit AT after transection and 4-strand Becker suture. (E) Cast with an ankle angle of 180 deg. (F) Opened wound site 3 weeks post-operation with repaired AT. (G) Extracted tendon 3 weeks post-operation.

The rabbits received premedication with 65 mg/kg body weight ketamine and 4 mg/kg Xylazine. A venous catheter was inserted in the marginal ear vein. The rabbits were intubated with propofol i.v. 0.6 mg/kg–1.3 mg/kg. Anaesthesia was maintained with 1–2% isoflurane (Fig. 4A). In order to ensure systemic analgesia during the time of operation, 0.2–0.3 mg/kg body weight Butorphanol (Dr. E. Graeub AG, Berne, Switzerland) was applied pre-operatively. The hind legs were shaved (Fig. 4B) and cleaned with iodine. The Achilles tendon exposure was obtained through a paratendineal incision of cutis, subcutis and fascia (Fig. 4C). The medial and lateral gastrocnemius of the Achilles tendon complex were then sliced perpendicularly to the length of the tendon 2.0 cm above the calcaneus and the two tendon stumps were sutured (4-strand Becker suture) using a USP 4.0 polypropylene thread (Fig. 4D). Subsequently the wound was closed with a running suture (using a USP 6.0 polypropylene fiber) of the fascia and interrupted skin. Immediately post-surgery, a Durogesic Matrix patch (Janssen-Cilag AG, Switzerland) was applied with 4.2 mg Fentanyl per patch in order to provide analgesia for about 72 h with 25 µg/h Fentanyl.

Postoperative treatment included a cast having an angle of 180 deg at the ankle for a total of 15 rabbits (adhesion provoking immobilization) (Fig. 4E). The angles at the ankle (from the knee to the ankle and from there to the toes) were measured with a goniometer. The cast was well padded: after application of Bactigras^®^ (Smith & Nephew, Austria), Elastomull^®^ (BSN Medical, Germany) and cohesive bandage, a stocking was pulled over. Then Terry Pad (Hauser Medizintechnik gmbh, Austria) was used to pad it dorsally and ventrally. After a base coat with soft bandage, a Scotchcast™ conformable splint (3M Medica AG, Switzerland) dorsally as well as ventrally was applied. The cast was changed after 3 weeks and removed after another 3 weeks for rabbits that were in the experiment for 6 weeks; for the 3-week and 12-week group the angle was also 180 deg, however, it was removed after 3 weeks and not changed further. For the other nine rabbits, the cast was also changed after 3 weeks; however, a cast with a smaller angle of 150 deg was applied (adhesion inhibiting immobilization). Great attention was paid to make the casts not too tight so that it was tolerated well by the rabbits (they did not bite the cast). Considering further control groups, there was no group that had their tendons operated but no cast applied, because this would lead to re-rupture immediately; and there was no group either that was not operated (untreated) and got an immobilization. 3, 6 or 12 weeks post-surgery, the rabbits were euthanized in deep anaesthesia (100 mg/kg Ketamine and 4 mg/kg Xylazine) with 80 mg/kg Pentobarbital (Esconarkon *ad us. vet*., Switzerland) and the tendons were extracted (Fig. 4F and G).

### Treatment groups

**Fig. 5.**
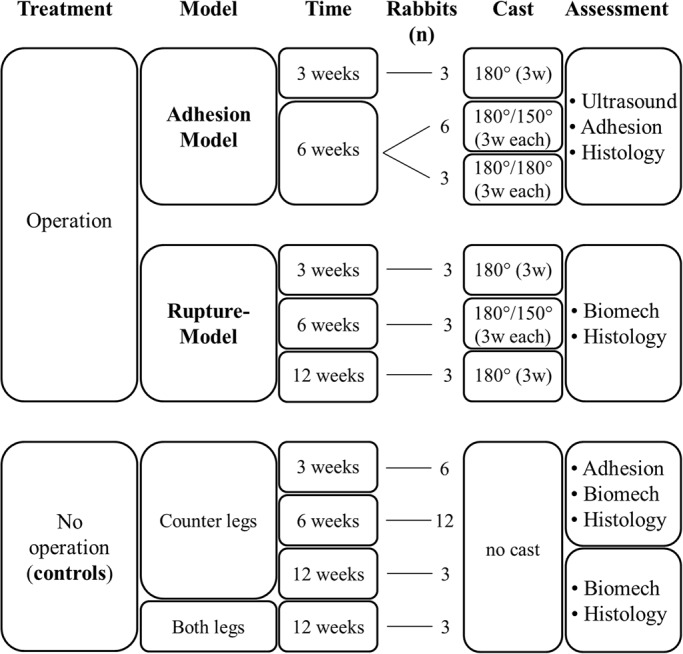
**Experimental groups depicting time point of tendon extraction, number of rabbits used for the corresponding assessments and post-operative treatments.** Accordingly, the two different approaches are given (Ultrasound, determination of adhesion gliding functionality by dynamic ultrasound; Adhesion, determination of adhesion extent by histological analyses; Histology, samples used for histopathological scoring after Stoll et al. (2011); Biomech, determination of load until failure, cross-sectional area, stiffness, failure stress and elastic modulus). The three different casting regimens were: 180 deg (3 w), a cast with an angle of 180 deg applied for 3 weeks, no further cast after that; 180 deg/180 deg, a cast with an angle of 180 deg applied for 3 weeks, after removal of first cast, another cast with an angle of 180 deg applied for another 3 weeks; 180 deg/150 deg, a cast with an angle of 180 deg applied for 3 weeks, after removal of first cast, another cast with an angle of 150 deg applied for the subsequent 3 weeks.

The 24 rabbits were randomly distributed into seven groups with *n*=3 or 6 (Fig. 5). All were operated on one hind leg except for three rabbits that were not operated at all (unbiased control group) (Buschmann et al., 2013a,b). The tendons of 12 rabbits were analysed histologically for adhesion scoring at two different healing times: three rabbits at 3 weeks post-operation after application of a cast of 180 deg; nine rabbits at 6 weeks post-operation (six rabbits in the 180 deg/150 deg and three in the 180 deg/180 deg group) where the angle groups were classified by the casting protocol [ankle angle change from 180 deg to 150 deg (180 deg/150 deg group) or 180 deg for the full 6-week period with a cast change after 3 weeks (180 deg/180 deg group)]. The tendons of 12 rabbits were analysed biomechanically (nine having a 4-strand suture, three not treated at all): three rabbits were analyzed after 3 weeks post-surgery receiving a 180 deg immobilization; three rabbits 6 weeks post-surgery receiving a 180 deg/150 deg immobilization; and six rabbits after 12 weeks post-surgery receiving a 180 deg immobilization, including three animals with no operation at all (serving as unbiased controls). The counter hind legs of all operated animals were not operated, not treated (NT), and served as control.

### Quantification of adhesion extent

After extraction, the Achilles tendon specimens were immediately frozen at −20°C. After being thawed to room temperature, they were dehydrated, paraffin-embedded and sectioned into 5 µm thick slices, which were cross-sections in the wound region (perpendicular to the Achilles tendon). After de-paraffinizing with xylene and rehydrating the sections, they were stained with Hematoxylin-Eosin (H&E), Alcian Blue (AB) and Picrosirius Red (PS) according to commonly established procedures.

Picrosirius Red stained sections were used to quantify the adhesion extent at 8× magnification (Leica EZ4D microscope, Switzerland). Here, adhesion formation was quantified in five subsequent cross-sections separated by 2.0 mm using a method by Tan et al. (2010). The percentage of adhesion was calculated by the length of the contact region of the tendon under view with the surrounding tissue divided by the total perimeter. The length of the contact region and the whole perimeter were determined using synedra View 3 software (version 3. 1. 0. 3.).

### Histopathological characterization

H&E and AB stained sections were used to apply a histopathological scoring system developed by Stoll and co-workers (2011). In each histological section, five fields of view (FOV) at 25× and 100× magnification, respectively, were analysed with a light microscope (Leica DM 6000 B) equipped with a digital camera. This system included inspection of the extracellular matrix organization of the whole repaired tendon (scores 0, 1 and 2), cellularity (scores 0, 1 and 2), cell alignment (scores 0, 1 and 2), cell nucleus morphology (scores 0, 1 and 2), cell distribution (scores 0 and 1), organization of the tendon callus (scores 0, 1 and 2), configuration of callus (scores 0 and 1 based on macroscopic observation of the specimen), integration of constructs to the normal tissue (scores 0, 1 and 2), vascularization (scores 0 and 1), metaplasia (scores 0, 1, 2 and 3) and features of inflammation (scores 0 and 1). For the proteoglycan content scoring (scores 0 and 1), AB-stained sections were used to check whether sulphated proteoglycans were present. Here, 5 FOV of each object were semi-quantitatively analysed. The scores were summed up (maximum 20), with 0 scores standing for damaged tissue and 20 scores for healthy tissue. For details of specific scores and exemplary histological images, see Table 1.

### Dynamic ultrasound

Ultrasound imaging was performed with an ultrasound unit (iU22 Ultrasound System, Philips Healthcare, Switzerland) with a linear high-frequency hockey-stick probe of 17.5 MHz (L17-5io Broadband Compact Linear Array Transducer, Philips Healthcare, Switzerland). The examination protocol consisted of a dynamic imaging of the gliding Achilles tendon by moving the flexor digitorum superficialis of the rabbit paw. During analysis, the gliding was scored with 0=good gliding (no adhesion), 1=middle gliding (some adhesion) and 2=no gliding (maximum adhesion). Color gain adjustment was calibrated on the counter leg that was not treated (healthy side).

### Biomechanical tests

Before measurement at room temperature (21°C), the tendons were thawed overnight at 4°C. The suture threads were not removed. All tendons were harvested from the hind legs including the muscle and the calcaneus. On the muscle side, the samples were mounted in serrated clamps after being wrapped in two pieces of cloth to reduce slippage (Rigozzi et al., 2009), and on the bone side, a device fixing the calcaneus in a rectangular position to the tendon was used (meaning a 90 deg angle between calcaneus and the stretched tendon). All samples were tested in uniaxial tension to failure at 1 mm/min speed on a universal material testing machine (Zwick 1456, 1 kN load-cell, TestXpert 10, Germany) with preconditioning (10 cycles to 10 N). The samples were sprayed with phosphate buffered saline during measurement in order to prevent drying. Load until failure (N) was determined as the maximum load measured.

The CSA was determined 2.0 cm above the calcaneus by a custom designed linear laser scanner adapted by Vergari et al. with *n*=6 per specimen before tensile testing (Vergari et al., 2010; Fessel et al., 2014). The load until failure (N) was divided by the thus-determined CSA at the repair site (mm^2^) resulting in the failure stress at the repair site (MPa). The elastic modulus (E-Modulus; MPa) was determined as the slope in the stress-strain curves.

### Statistical analysis

Adhesion extent, histopathological scores and biomechanical data were analysed with StatView 5.0.1 (SAS Software). Either unpaired *t*-test was used to compare operated and untreated specimen, or one-way analysis of variance (one-way ANOVA) was conducted to compare three situations as found for two different casting regimens and the untreated specimen. Pairwise comparison probabilities (*P*) were calculated using the Fisher's PLSD. *P*-values <0.05 (*) were considered significant; **P<0.01; ***P<0.001. Values were expressed as means±standard deviations.
